# Characterization of Turner Syndrome-associated Diabetes Mellitus

**DOI:** 10.1210/clinem/dgae357

**Published:** 2024-07-04

**Authors:** Antoinette Cameron-Pimblett, Clementina La Rosa, Melanie C Davies, Jenifer P Suntharalingham, Miho Ishida, John C Achermann, Gerard S Conway

**Affiliations:** Reproductive Medicine Unit, University College London Hospital, London, WC1N 6HU, UK; Reproductive Medicine Unit, University College London Hospital, London, WC1N 6HU, UK; Reproductive Medicine Unit, University College London Hospital, London, WC1N 6HU, UK; Genetics & Genomic Medicine Research and Teaching Department, UCL Great Ormond Street Institute of Child Health, University College London, London, WC1N 1EH, UK; Center for Endocrinology, Charterhouse Square, Barts and the London School of Medicine and Dentistry, Queen Mary University of London, London, EC1M 6BQ, UK; Genetics & Genomic Medicine Research and Teaching Department, UCL Great Ormond Street Institute of Child Health, University College London, London, WC1N 1EH, UK; Reproductive Medicine Unit, University College London Hospital, London, WC1N 6HU, UK

**Keywords:** Turner syndrome, diabetes mellitus, autoimmunity, glucose homeostasis, SNVs, genetic variability

## Abstract

**Context:**

Diabetes mellitus (DM) risk factors in Turner syndrome (TS) may include autoimmunity, obesity, β-cell dysfunction, genetic predisposition, and insulin resistance (IR).

**Objective:**

This work aimed to evaluate glucose tolerance and DM risk factors in adults with TS.

**Methods:**

A single-center study with 2 phases was conducted to determine the prevalence of DM and to assess DM risk markers comparing women with TS with and without impaired glucose tolerance (IGT). The study took place at a tertiary referral center, University College Hospitals. A total of 106 women with TS (age range, 18-70 years) undergoing annual health surveillance underwent oral glucose tolerance tests (OGTTs), with additional samples for autoimmunity and genetic analysis. Main outcome measures included glucose tolerance, insulin, autoimmune, and single-nucleotide variation (SNV) profile.

**Results:**

OGTT screening showed that among those without a previous DM diagnosis, 72.7% had normal glucose tolerance, 19.5% had IGT, and 7.6% were newly diagnosed with DM. OGTT identified more cases of DM than glycated hemoglobin A_1c_ sampling alone. Women with IGT or DM were older, with higher body mass index and IR. No association was found between autoimmune markers glutamic acid decarboxylase (GAD), islet antigen-2, and zinc transporter 8, risk karyotypes, or selected SNVs and DM. In DM cases, GAD positivity was associated with requirement for insulin therapy. The median age of onset of the diagnosis of DM was 36 years (range, 11-56 years).

**Conclusion:**

In the spectrum of DM subtypes, TS-associated DM lies between type 1 and type 2 DM with features of both. Key factors include weight and IR. Assessing C-peptide or GAD antibodies may aid future insulin requirement.

Turner syndrome (TS) is one of the most common chromosomal anomalies (1). Among the many comorbidities associated with the lack of X chromosome material is diabetes mellitus (DM). There are several features of TS that could contribute to an excess risk of DM including autoimmunity, obesity, β-cell dysfunction, and insulin resistance (IR) but so far, the relative importance of each of these is poorly understood.

Data on the prevalence of established DM in women with TS have been estimated using various methodologies. A sex-matched registry data analysis estimated DM risk in TS to be raised 4-fold in the case of type 1 DM (T1DM) and 2-fold for type 2 DM (T2DM) (2). Screening studies using oral glucose tolerance tests (OGTTs) in study populations have reported an incidence of T2DM between 12% and 13% (3, 4). Relatively few cases of T1DM have been well characterized, and the label has been used mainly according to age of onset rather than autoimmunity (3, 5).

Studies of glucose homeostasis in women with TS have shown variable results. Insulin resistance (IR) has been reported in adolescents (6) but less so in adults (3, 7). IR, when identified, appears to be independent of obesity but may be related to the timing of induced puberty (6). With regard to insulin secretion in TS, reduced first-phase insulin response has been demonstrated using either OGTTs (3, 4) or intravenous GTTs (8).

Autoantibodies have been used to differentiate diabetes subtypes (9). The most commonly used antibodies are associated with pancreatic β-cell destruction and reduce insulin secretion downstream: glutamic acid decarboxylase (GAD), zinc transporter 8 (ZnT8), and islet antigen-2 (IA-2). DM-related autoimmunity has been reported in several cohorts of women with TS but in only a few women with established DM. GAD positivity is found in approximately 3% of women with TS compared to 1% in the general population (5, 10, 11). In women with TS and DM, however, an excess prevalence of DM-associated autoantibodies has not been reported (3, 4).

The mechanism by which X chromosome anomalies lead to DM is uncertain but some karyotype subgroups are thought to be at particular risk. Salgin et al (6) reported an association between the 45,X karyotype subgroup and DM risk factors, which is to be expected as this subgroup can present with a more severe phenotype compared to mosaic forms. Further, the isochromosome and ring chromosome subgroups have been reported to be associated with DM risk when compared to the 45,X subgroup, believed to be the result of abnormal gene dosing (3, 12, 13).

The pathogenic pathways of DM in TS remain to be clarified. Previous studies have been conflicting with varying methodologies and cohort sizes. Here, we present data from a prospective screening study using OGTTs followed by targeted characterization of women with TS and established DM to clarify the aforementioned observations.

## Materials and Methods

This study formed part of the Reproductive Life Course Project and was approved by the Chelsea Research Ethics Committee (16/LO/0682) and covers a range of investigations and retrospective audits that have been published previously (12, 14-18). Women attend the Adult Turners Clinic at University College London Hospital (UCLH) on an annual basis from which participants in these studies were recruited prospectively. We sought to identify the prevalence of impaired glucose tolerance (IGT) and DM in TS in addition to characterizing the DM phenotype both in established and newly diagnosed DM individuals. Records from the Adult Turner Clinic at UCLH were used to identify women whose clinical and genetic profile fulfilled criteria for 4 substudies each focusing on specific questions regarding the etiology of DM in TS. Participation in the 4 studies was not mutually exclusive as the clinical profile of many women with TS fulfilled criteria for more than 1 study. For instance, those diagnosed with IGT or DM in study 1 were included in study 3.

### Participants

#### Oral glucose tolerance test screening in women without previously diagnosed diabetes mellitus

An unselected group of women with TS with varied karyotype who had not previously been diagnosed with IGT or DM were invited to attend for an OGTT as part of their routine annual review with the aim of estimating the prevalence of occult abnormalities of glucose homeostasis in our clinic population.

#### Comparison of prevalence of impaired glucose tolerance/diabetes mellitus status in karyotype subgroups

To clarify the association between DM status and karyotype, 3 subgroups were sought for targeted recruitment: (i) 45,X; (ii) nonmosaic isochromosome 46,X, i(Xq); and (iii) mosaic ring chromosome 45, X/46,X,r(X) (3, 6, 12, 19). The nonmosaic isochromosome group were selected as opposed to mosaic 45,X/46X,i(Xq) as they should have the greatest degree of gene dosage effect of X chromosome genes. Ring X exists only in mosaic form. An OGTT was performed for those without previously diagnosed DM, and a fasting blood sample was drawn for women with established DM.

#### Glucose homeostasis and autoimmunity characterization of women with Turner syndrome and abnormal glucose tolerance

Women with TS and established DM were invited to attend to characterize the clinical and autoimmune features of TS-associated DM. Twenty-nine (52.7%) of 55 women who were recorded as having established DM on UCLH records were recruited. The remaining 26 of 55 (47%) were unable to attend within the time frame of this study or no longer attended the clinic. In this group with DM, a blood sample was drawn and tested for plasma glucose concentration and insulin after 10 hours of fasting before the administration of antidiabetic medications. Blood samples were used to test for GAD (RRID: GDE/96), IA-2 (RRID: IAE/96/2), and ZnT8 (RRID: ZnT8/96) autoantibodies. Data from this group were combined with newly diagnosed IGT/DM from studies 1 and 2.

#### Pilot study of diabetes mellitus–related single-nucleotide variations in women with Turner syndrome

DNA extracted from whole blood was collected from individuals in the first 2 studies for a pilot assessment of single-nucleotide variations (SNVs; formerly single-nucleotide polymorphisms [SNPs]) known to be associated with type 2 DM (T2DM) in population genome-wide association studies (GWAS) (20, 21). Women with newly diagnosed IGT/DM (study 1) or established DM (study 2) were combined as a single group and compared to women with normal glucose tolerance. In this case-control design of varied karyotype backgrounds, cases were defined as having established DM or newly diagnosed IGT or DM (n = 45). Controls were defined as having proven normal glucose tolerance with an OGTT (n = 56).

### Establishing Reference Ranges for Outcome Parameters in Women With Estrogen Deficiency

Fifteen women with primary ovarian insufficiency (POI) with a history of primary amenorrhea and normal 46,XX karyotype were recruited to establish reference ranges for baseline biochemical parameters in women with estrogen deficiency whose age and type of exogenous estrogen were similar to women with TS. These women attended for a single fasting blood sample.

#### Clinical assessment

The assessment visit comprised a record of height, weight, waist circumference (cm), and fat mass (Tanita Body Composition Analyzer BC-418 MA). The variable body mass index (BMI) was correlated closely with waist circumference (*r* = 0.86) and percentage body fat (*r* = 0.90), and neither of these variables had an independent influence of subsequent analyses. BMI was chosen pragmatically to represent the effect of body weight.

At baseline, blood samples were drawn for glucose, insulin (Abbott Clinical Chemistry Autoanalyzer), and C-peptide (AutoDELFIA kit). Homeostatic Model Assessment of Insulin Resistance (HOMA-IR) was calculated using the following: fasting insulin (mU/L) × fasting glucose (nmol/L)/22.5. IR was defined as a calculated IR above 2.5. The C-peptide reference range was 260 to 650 pmol/L. The DM-related autoantibodies GAD, IA2, and ZnT8 were measured by the UK regional center in Exeter (11). Positive titer threshold for autoantibodies from this laboratory were GAD+ greater than or equal to 11 U/mL, IA-2+ greater than or equal to 7.5 U/mL, and ZnT8+ greater than or equal to 65 U/mL for individuals aged 30 years or younger or ZnT8+ greater than or equal to 10 U/mL for older participants.

The screening study was performed using 75-g OGTT after 10 hours of fasting with samples taken at 0, 60, and 120 minutes. IGT was defined as a fasting plasma glucose of 6.1 to 6.9 mmol/L or 7.8 to 11.1 mmol/L after 120 minutes and DM as glucose of 7.0 or greater or 11.1 mmol/L or greater, respectively.

### Single-Nucleotide Variation Genotyping and Association Analysis

Previously reported T2DM-associated SNVs were selected for genotyping based on having sufficient minor allele frequency (> 0.25) and odds ratio for risk (OR > 1.05) to have the power to allow for small cohort assessment. A total of 45 different SNVs were analyzed using leukocyte DNA and 2 different approaches in a core group of 101 women (Supplementary Table S1) (22).

1. Infinium Global Screening Arrays (v3.0 A1, Illumina) were undertaken and data outputted into GenomeStudio Genotyping Module software (v2.0, Illumina) for genotype calling and quality control, using hg19 genome build manifest (GSA-24v3-0_A2) containing 654 027 SNVs. All samples had an SNV call rate above 97%. The genotypes of 22 SNVs with known associations with T2DM were exported for further analysis.
2. An additional 24 SNVs of interest that were absent in the SNV array were genotyped with custom whole-exome sequencing by inclusion of custom probes on the Nonacus ExomeCG design (Nonacus). Binary alignment map files were produced by aligning FASTQ files to the GRCh38 reference genome with Burrows-Wheeler Aligner (v0.7.17). Read annotation and grouping by unique molecular identifiers were performed with fgbio (v0.4.0). Genotypes were extracted with bcftools mpileup function (Samtools v1.9) to produce a genomic variant call format (GVCF) file, which additionally includes the homozygous reference genotypes to a standard VCF format specification. Resulting GVCF files were tabulated with the Genome Analysis Toolkit (GATK) VariantsToTable tool (v4.1.8.1). One of the SNVs (rs6780171) was removed due to its low call rate (<97%). A combined total of 45 SNVs was used for further analysis (Supplementary Table S2) (22).

SNV association tests were performed with the SNPassoc package (v 2.0.11) in R (v 4.0.3). TS women with (n = 45) and without (n = 56) DM were used as cases and controls, respectively. None of the SNVs in the control samples deviated from Hardy-Weinberg equilibrium test (*P* < .05). The SNVs were tested for association with DM using logistic regression analysis, with and without adjustment for age and BMI, under an additive model. The SNVs were additionally tested for association with BMI alone with linear regression analysis under an additive model. The genomic inflation factor λ (lambda) was estimated with the GenomicControl function.

### Statistical Analyses

Two statistical analyses were used to test for group differences in the characterization of DM in TS using SPSS version 25 (SPSS Inc). Continuous variables were tested by analysis of variance with supplementary Tukey post hoc analysis when required. Categorical variables such as antibody status were tested by chi-square analysis. Binary regression was used to assess the relationship between DM status and risk factors BMI and HOMA-IR, controlling for age. A nonparametric Kruskal-Wallis test was used for biochemical parameters and interquartile ranges calculated.

## Results

### Oral Glucose Tolerance Testing Screening in Women Without Previously Diagnosed Diabetes Mellitus

OGTTs were performed in 77 women with TS without previously diagnosed DM; 56 (72.7%) participants had normal glucose tolerance, IGT was diagnosed in 15 of 77 (19.5%), and newly identified DM in 6 of 77 (7.6%). For comparison, glycated hemoglobin A_1c_ (HbA_1c_) was less than 5.7% in 67 (87%), between 5.7% and 6.4% in 9 (11.7%), and greater than 6.4% in 1 (1.3%); (*P* = .048 compared to OGTT by chi-square 3-way analysis). The cutoff of HbA_1c_ greater than 5.7% failed to identify 11 of 21 (52.4%) cases of abnormal glucose tolerance.

### Comparison of the Prevalence of Impaired Glucose Tolerance/Diabetes Mellitus Status in Key Karyotype Subgroups

Table 1 shows the assessment of DM risk against previously identified risk karyotypes: 17 women with nonmosaic isochromosome X—46,X,i(Xq), and 22 women with mosaic ring X—45, X/46,X,r(X). Chi-square analysis was performed comparing the prevalence of IGT/DM in these groups separately with 53 women with monosomy X (45,X). The prevalence of IGT/DM in the 45,X group was not statistically different from the prevalence in either the ring chromosome or isochromosome groups.

**Table 1.** Prevalence of normal glucose tolerance vs impaired glucose tolerance/diabetes mellitus for the ring chromosome and isochromosome karyotype groups each compared to that of 45,X by *t* test and chi-square analysis

	45,X	46,X,i(Xq)	45,X/46,X,r(X)	*P*
No.	53	17	22	—
Age (mean [SD]), y	33.3 (11.6)	30.5 (12.1)	31.7 (10.7)	.61
BMI (mean [SD])	27.4 (6.2)	26.3 (7.4)	25.5 (5.6)	.58
Normal glucose tolerance	52.8%	70.6%	68.2%	.83
Impaired glucose tolerance or diabetes	47.2%	29.4%	31.8%	.83

Abbreviation: BMI, body mass index.

To test a possible effect of gene dosing on autoimmune DM risk in women with isochromosome variants, we specifically assessed DM-related autoimmune markers in 11 women with nonmosaic 46,X,i(X) and 6 with mosaic 45,X/46,X,i(X). In the former group there was 1 positive for GAD but no one positive for IA2 and Z1T8. In the mosaic isochromosome group, there was 1 positive for GAD, 1 positive for IA2, but no one positive for Z1T8. These changes were not significant when compared to data from women with 45X karyotype.

In summary, we found that the 2 karyotype subgroups of isochromosome X and ring X were not associated with an excess risk of DM or DM-related autoimmunity, but the numbers in these subgroups were small.

### Glucose Homeostasis and Autoimmunity Characterization of Women With Turner Syndrome and Abnormal Glucose Tolerance

Table 2 shows the results of the anthropometric, biochemical, and autoimmunity parameters comparing TS women who had normal glucose tolerance with those with IGT or DM (including established DM). In addition, data from nondiabetic women with POI was compared to TS women and normal glucose tolerance. Estrogen replacement therapy (ERT) use by the 106 women with TS was as follows: forms of oral estradiol 68 (64%) women, transdermal estradiol 13 (12%), combined oral contraceptive pill 7 (7%), no ERT (stopped after age 50 years) 13 (12%), no ERT because of spontaneous periods 4 (4%). After correcting for age and DM status, there was no association between ERT subgroup and parameters of glucose homeostasis. Regarding endogenous estrogen status, 89 (84%) women experienced primary amenorrhea, 12 (11%) secondary amenorrhea, and 4 (4%) had ongoing regular cycles. There was no significant difference in mean insulin, glucose, or HOMA-IR results between these groups.

**Table 2.** Clinical, biochemical and autoimmune characteristics in individuals grouped by diagnosis and diabetes status

	POI	TS Normal glucose tolerance	TS IGT and DM
No.	15	56	50
Age, y	31 (±11.1)	32.1 (±10)	39.3 (±12.4)*^a,d^*
Anthropometrics			
BMI	24 (±6.2)	25.6 (±6.2)	29.7 (±6.6)*^a,d^*
Waist Circumference, cm	83.9 (±20.6)	84.1 (±13.4)	95.6 (±15.4)*^a,d^*
Body fat, %	31.2 (±9.6)	27.9 (±11.2)	33.2 (±9.6)*^a,c^*
Biochemical markers			
Fasting glucose, mmol/L	4.5 (4.4-4.8)	4.4 (4.2-4.7)	5.4 (4.5-7.4)*^a,d^*
Fasting insulin, pmol/L	8.2 (6.4-10.7)	5.5 (4.1-7.2)	7.2 (3.6-10.6)*^a,d^*
C-peptide, pmol/L	506 (453-566)	422 (322.8-533)	682 (450-1266)*^a,d^*
HbA_1c_, %	5.3 (5.0-5.4)	5.2 (4.9-5.5)	5.8 (5.3-8)*^a,d^*
HOMA-IR > 2.5	27%	6%*^b,c^*	52%*^a,d^*
Autoimmunity markers			
TPO+	7.7%	42.5%*^b,c^*	42.5%
GAD+	0%	3.8%	15.9%
IA-2+	0%	3.8%	9.4%
ZnT8+	0%	3.9%	2.3%

Data presented as mean (SD) for anthropometric measures, and median, interquartile range biochemical outcomes, and percentage of positive autoantibodies. Individuals with IGT and DM cases have been combined for the purpose of analysis.

Abbreviations: DM, diabetes mellitus; GAD, glutamic acid decarboxylase; HbA_1c_, glycated hemoglobin A_1c_; HOMA-IR, Homeostatic Model Assessment of Insulin Resistance; IA-2, islet antigen-2; IGT, impaired glucose tolerance; POI, premature ovarian insufficiency; TPO, thyroid peroxidase; TS, Turner syndrome; ZnT8, zinc transporter 8.

^*a*^Different from TS normal glucose tolerance group.

^*b*^Different from POI group.

^*c*^ *P* less than .05.

^*d*^ *P* less than or equal to .01.

Regarding women with TS, those with IGT/DM were older with greater waist circumference and BMI (*P* < .01, respectively), higher body fat mass (*P* = .03), and had significantly greater mean glucose, insulin, and C-peptide concentrations compared to women with TS and normal glucose tolerance. IR defined by HOMA-IR greater than 2.5 was found in 52% of women with TS-associated DM compared to 27% of women with POI and 6% of women with TS and normal glucose tolerance. Regression analysis was performed to examine the influence of BMI and HOMA-IR in DM. BMI and HOMA-IR were both found to be independently associated with IGT/DM (partial β = 0.3 and 0.2, respectively; *P* < .01).

There was no significant association between thyroid peroxidase (TPO), GAD, IA-2, or ZnT8 positivity and DM risk in women with TS. When examining autoantibody status in more detail, there was a trend of GAD positivity in the IGT/DM group (15.9% vs 3.8%; *P* = .058) compared to those with normal glucose tolerance but not so for IA-2 or ZnT8. There were no differences in age or BMI comparing women who were GAD positive to those who were negative. Notably, 3 of 6 (50%) DM individuals who used insulin therapy were GAD positive compared to 3 of 16 (18.7%) participants who took oral antidiabetic treatments (*P* ≤ .01). Moreover, women with established DM and GAD positivity were found to have significantly reduced C-peptide concentrations compared to their GAD-negative counterparts (201.1 pmol/L vs 1010.4 pmol/L; *P* ≤ .01).

**Figure 1.**
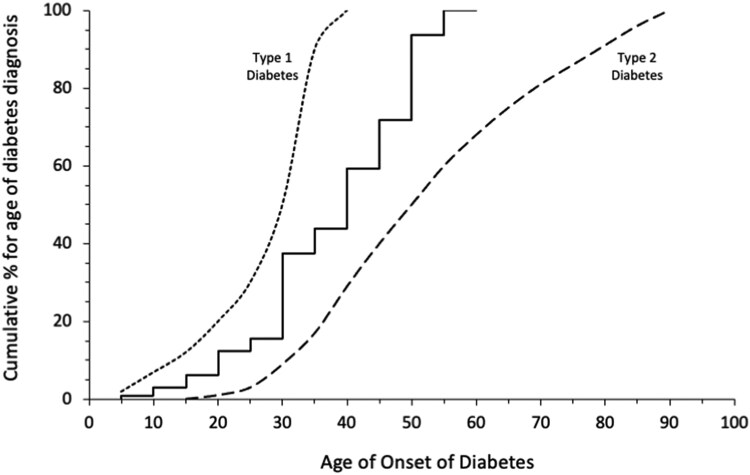
Age of onset of Turner syndrome–associated diabetes mellitus. The age of onset of diabetes mellitus in this cohort of women with Turner syndrome (solid line) compared to representative population data on the age of onset of those with type 1 diabetes (dotted line) and type 2 diabetes (dashed line) (23).

Regarding the 29 women with TS and an established diagnosis of DM, the median age of diagnosis of DM was 37 years (range, 11-56 years). Fig. 1 shows that the cumulative frequency of the age of onset for those with new and established TS-associated DM was midway between a representative age of onset of T1DM and T2DM derived from published data (24). Median duration of DM was 8.9 years (range, 7 months-25 years). DM treatment for women with established DM was recorded as diet alone 2 of 29 (6.9%); oral antidiabetics 21 of 29 (72.4%); insulin therapy 6 of 29 (20.7%), including 1 of 29 (3.4%) using insulin and oral antidiabetics combined.

### Pilot Study of Diabetes Mellitus–Related Single-Nucleotide Variations in Women With Turner Syndrome

There was no evidence of strong associations between the selected SNVs and T2DM or BMI in this cohort (Supplementary Table S2, Supplementary Figs. S1 and S2) (22). rs4886696, an intron variant of *SIN3A*, showed nominal significance with DM (*P *< .05), with or without adjustment for age and BMI, but not after Bonferroni correction. rs10830963, an intron variant of *MTNR1B*, showed nominal significance with a DM association but it no longer showed association after correction for age and BMI. rs1553921 and rs1124777, synonymous variants of *PIK3C2B*, showed nominal significance with BMI.

## Discussion

In this study, we investigated glucose homeostasis and possible pathogenic mechanisms for DM in women with TS with and without DM. Abnormal glucose tolerance was identified in 27.3% of women with TS who had not previously been diagnosed. OGTT identified significantly more cases than a single HbA_1c_ test. DM in women with TS is associated with IR that is not fully accounted for by weight, and IR may be an innate feature of TS prior to the onset of DM. DM-related autoimmunity and the isochromosome X and ring X karyotypes were not significant risk associations for DM in women with TS. The age of onset of DM in women with TS was found to fall midway between population references for T1DM and T2DM, which could indicate it being a distinct entity or a mix of the 2 conditions.

In the screening study with OGTT, we found IGT in 19.5% and T2DM in 7.8% of women with TS in whom DM had not been diagnosed by routine clinic assessment, which was comparable to previous studies that together showed a mean IGT risk of 23% and 13% (3, 4). The study by Ibarra-Gasparini et al (4) added the expected finding that IGT progresses to DM even after a relatively short follow-up interval. Our finding that HbA_1c_ did not perform as well as OGTT in identifying cases with DM is in agreement with several studies that compared the 2 tests (25, 26). The 2 tests, however, were similarly effective in predicting the progression to DM complications such as retinopathy in the DIRECT-2 project (27). The average age of onset of 36.1 years in our cohort is also similar to the aforementioned studies and follows a similar pattern to that found in Latent Autoimmune Diabetes in Adult (LADA) (9, 28, 29), as shown in Fig. 1. Like LADA, TS-associated DM appears to be on a spectrum between the 2 very different disorders of glucose homeostasis, T1DM and T2DM. In Table 3 we compare selected features of the different types of DM and show similarities with LADA.

**Table 3.** Comparison of characteristics of 3 subtypes of diabetes compared to the current study of TS associated DM

Features	T1DM	T2DM	LADA	TS-associated DM
Age of Onset ± SD	12.6 ± 1.34	48.0 ± 0.5	33.4 ± 2.15	35.8 ± 10.9
Prevalence of GAD	85%	7%	68.6%	22.2%
Insulin Resistance	32%	89%	42%	52%
Insulin therapy	Always	Sometimes	Eventually	20.7%
C-peptide	Low	High	Variable	High
Inheritance pattern	Sometimes	Yes	Yes	Sporadic
Obesity related	No	Yes	Yes	Yes
Associated Autoimmunity	Yes	No	Yes	Yes

Reference data were extracted from a variety of sources (9, 30-32).

We confirmed a finding of IR manifested by raised fasting insulin and HOMA-IR in TS-associated DM compared to nondiabetic counterparts (4, 6, 8). Interestingly, IR associated with DM was not completely accounted for by weight as HOMA-IR was significantly raised even when controlled for BMI. While IR is a cardinal feature of T2DM, a recent study found that LADA participants with low GAD titers had an increased prevalence of IR (33).

In common with other forms of autoimmunity, we found that women with TS were 8.5 times more likely to be GAD positive compared to the general population (11). When observing women with DM alone, 22% were GAD positive vs 5.3% of a previous study (3). While the frequency of GAD positivity may not be as high as the estimated 68.6% of those with LADA (34), it is considerably higher than that of T2DM (31). The observation that GAD positivity was found to be associated with lower C-peptide makes it worth considering screening women with TS and DM for either GAD or C-peptide in women with TS and DM as an indicator of when insulin treatment may be required.

The isochromosome and ring chromosome karyotypes have previously been associated with DM and therefore we recruited these subgroups specifically. Moreover, to increase the likelihood of detecting a gene dosing in isochromosome X we focused on the nonmosaic form rather than the mosaic isochromosome, which predominated in earlier studies (3). We did not find an association between either karyotype and prevalence of IGT/DM when compared to 45,X (see Table 1). We conclude from this that the pursuit of genes for which triploid representation could be a risk of DM is unlikely to be a fruitful route to new pathogenic mechanisms in DM, accepting that the subgroups were small and analysis underpowered to provide a definitive conclusion.

Finally, analysis of 45 common prevalent SNVs that have been reported to be associated with T2DM in population GWAS studies did not show any clear positive relation to DM in the study population. Although these SNVs were chosen based on having a sufficiently high allele frequency to detect differences, the numbers studied are still potentially likely to be underpowered for definitive assessment. We estimate that around 250 diabetic and 250 nondiabetic TS women would be needed for conclusive assessment of the genetic contribution to DM in TS, depending on the minor allele frequency of the variant in the study population. Furthermore, the odds ratios (effect size) of even the most strongly associated SNVs in GWAS studies is still low, so it is likely that the contribution of common SNVs to the emergence of DM in TS is limited, as might be expected.

In a related study using whole-exome sequencing comparing women with TS with controls and women with POI, Suntharalingham et al (18) found that X-chromosome variants were proportionate to the complement of X-chromosome material across the groups and that no X chromosome gene/variants were strongly enriched in monosomy X women with DM compared to monosomy X women without. Therefore, unlike the association of *TIMP3* gene variance being associated with congenital cardiac anomalies that was replicated by Suntharalingham et al (18), DM does not have clear associations with genetic variants using this methodology (35).

Despite this being one of the largest series of women with TS diagnosed with DM, the conclusions from this observational study are limited in that subgroup analyses include comparisons that do not have sufficient power to enable definitive conclusions. In this regard, this study can be taken as pilot data to enable studies of greater power to reduce the chance of false negatives. However, some comparisons in this study, such as the analysis of karyotype subgroups, are hypothesis driven, providing balance to multivariate association studies that are more prone to false positives.

In conclusion, TS-associated DM could be a particular entity among the DM subtypes with a unique profile that encompasses elements of lifestyle risk factors, autoimmunity, and IR of unknown origin. On the spectrum of DM subtypes, TS-associated DM resides between LADA and T2DM. We found HbA_1c_ to lack sensitivity in the diagnoses of abnormal glucose tolerance and DM. From the ULCH cohort we estimate that one-fourth of all women with TS will have some form of abnormal glucose homeostasis, emphasizing the need for regular OGTTs, particularly in those with a BMI of 25 or greater. Implementing routine OGTTs may allow for more timely lifestyle interventions and lessen chances of abnormal glucose intolerance and/or DM. From our findings, should a diagnosis of TS-associated DM be made we would suggest testing for C-peptide or GAD in women, which could give an indication if the individual may require insulin therapy in the future. We hypothesize that 2 mechanisms might account for IR in women with TS that is not accounted for by weight. Exogenous estrogen, either in the timing of induced puberty or in the dose of adult treatment, is an insulin sensitizer and relative resistance to estrogen in women with TS is possible (36). Second, there remains the possibility of a genetic pathway with haploinsufficiency of X-chromosome genes affecting insulin action or through a downstream effect on autosomal DM risk factors that has yet to be identified by current methods.

## Data Availability

All data sets generated during and analyzed during the current study are not publicly available but are available from the corresponding authors on reasonable request.

## Abbreviations

BMI: body mass index
DM: diabetes mellitus
ERT: estrogen replacement therapy
GAD: glutamic acid decarboxylase
GWAS: genome-wide association studies
HbA_1c_: glycated hemoglobin A_1c_
HOMA-IR: Homeostatic Model Assessment of Insulin Resistance
IA-2: islet antigen-2
IGT: impaired glucose tolerance
IR: insulin resistance
OGTT: oral glucose tolerance test
POI: premature ovarian insufficiency
SNV: single-nucleotide variation
T1DM: type 1 diabetes mellitus
T2DM: type 2 diabetes mellitus
TPO: thyroid peroxidase
TS: Turner syndrome
UCLH: University College London Hospital
ZnT8: zinc transporter 8
